# Community preferred drug delivery approaches for pilot roll-out of a potential novel paediatric schistosomiasis treatment option in two endemic counties of Kenya: A mixed methods study

**DOI:** 10.1371/journal.pgph.0003221

**Published:** 2024-05-31

**Authors:** Janet Masaku, John M. Gachohi, Alice Sinkeet, Mary Maghanga, Florence Wakesho, Wyckliff Omondi, Nora Monnier, Peter Steinmann, Lisa Sophie Reigl, Isabelle L. Lange, Andrea S. Winkler, Sammy M. Njenga, Mary Amuyunzu-Nyamongo

**Affiliations:** 1 Eastern and Southern Africa Centre of International Parasite Control (ESACIPAC), Kenya Medical Research Institute (KEMRI), Nairobi, Kenya; 2 School of Public Health, Jomo Kenyatta University of Agriculture and Technology (JKUAT), Nairobi, Kenya; 3 African Institute for Health and Development (AIHD), Nairobi, Kenya; 4 Division of Vector Borne and Neglected Tropical Diseases (DVB/NTDs), Ministry of Health (MoH), Nairobi, Kenya; 5 Department of Epidemiology and Public Health, Swiss Tropical and Public Health Institute, Basel, Switzerland; 6 Department of Neurology, Center for Global Health, Technical University of Munich (TUM), Munich, Germany; 7 Department of Community Medicine and Global Health, Institute of Health and Society, University of Oslo, Oslo, Norway; Cheikh Anta Diop University: Universite Cheikh Anta Diop, SENEGAL

## Abstract

Treating preschool age children (PSAC) for schistosomiasis has remained a challenge due to lack of a pediatric formulation. In response to this unmet need, the Paediatric Praziquantel Consortium has developed a potential novel paediatric treatment option. In advance to its roll-out to follow regulatory response, we conducted a social science study to gather information on preferred drug delivery approaches to inform implementation. A cross-sectional study was conducted in eight villages in two purposively selected Kenyan counties. A questionnaire was administered on 690 parents/guardians of PSAC at household level. Preferred drug delivery approaches were analyzed using frequencies and proportions. We conducted key informant interviews with 17 opinion leaders and 28 healthcare workers, and 12 focus group discussions with parents/guardians of PSAC and 7 with community health volunteers (CHVs). Thematic analysis was performed on the qualitative data. Majority of the 690 respondents were women 594 (86.1%) with a mean age of 34.1 (SD = 11.3, min-max = 18–86). Community-based mass drug administration (cMDA) was the most preferred drug delivery method by 598 participants (86.7%), followed by health facility/fixed points by 398 participants (57.7%). Similarly, in the qualitative data participants indicated they prefer cMDA since the CHVs who would distribute the medication are familiar with households with PSAC and are trusted to explain the drug effects. Health facilities/fixed points were the second most preferred drug delivery approach, but some health workers we interviewed expressed concern about potential understaffing and overcrowding of facilities. Appropriate timing of the drug distribution, not to interfere with farming activities, was considered critical, irrespective of the approach used. All profiles of study participants preferred cMDA over the other delivery approaches due to the convenience of receiving drugs at home and providing explanations about the new drug. For positive outcomes, adequate planning, proper timings and community involvement are highly recommended.

## Introduction

Schistosomiasis is a parasitic waterborne infection and a neglected tropical disease (NTD) of considerable public health importance globally. It is caused by a blood fluke of the genus *Schistosoma* with more than 250 million people infected and 779 million at risk. The global burden in 2016 stood at 1.864 million disability-adjusted life years (DALY) [1,2]. More than 90% of all schistosome infections are concentrated in Sub-Saharan Africa [3,4]. Preventive chemotherapy (PC) has been used for morbidity control, emphasizing periodic administration of praziquantel (PZQ) to at-risk populations without prior diagnosis. Distribution campaigns aim to achieve at least 75% of treatment coverage among school-aged children (SAC) in schistosome-endemic areas [5]. However, guidelines indicate that these measures be integrated with other supportive interventions, including provision of clean water, improvement of sanitation, and health promotion activities [6].

In Kenya, schistosomiasis is caused by two main species, *Schistosoma mansoni* and *Schistosoma haematobium*. It is estimated that more than six million people in the country, or approximately 23% of the population, are infected with either intestinal or urogenital schistosomiasis, and approximately 17.4 million people are at risk of getting infected [7]. The parasitic disease occurs mostly in western and coastal Kenya and selected foci in central Kenya. In coastal Kenya, schistosomiasis is exclusively caused by *S*. *haematobium*, while in the western region both species are prevalent [8]. Socio-economic status, human behaviour, environmental factors and demography may be partly responsible for the distribution of this disease [9].

While SAC are the main target population for treatment, it is becoming increasingly clear that younger children (<5 years) are also affected by schistosomiasis and suffer from morbidity as shown in previous studies in Côte d’Ivoire the prevalence was 25%, Tanzania 21.8% and in Kenya 17% [10–12]. Treatment of PSAC has been hampered due to the lack of a child-friendly treatment option for use in mass drug administration (MDA) programs [13]. In 2010, the World Health Organization (WHO) recommended extending large scale treatment programmes for schistosomiasis to PSAC ≥ 2 years of age [14]. In the absence of an appropriate paediatric formulation, broken or crushed praziquantel tablets have been recommended [15].

In response to the need for a suitable and safe drug formulation for this age group, the Pediatric Praziquantel Consortium (PPC) has developed a novel child-friendly orodispersible tablet (arPraziquantel 150 mg or arPZQ-ODT) [16]. Compared to the available praziquantel 600 mg tablets, the palatability of the new tablet has been improved [17] and it can be dissolved in liquid (2 ml per 150 mg tablet) thus facilitating more accurate and easier dosing for PSAC drug administration [18]. The drug has gone through clinical trials including Kenya, proved to be safe for use and will be rolled out in a pilot MDA [19].

Mass drug administration with PZQ is the main approach adopted by the Kenyan National School Based Deworming programme (NSBDP) for schistosomiasis control to reduce related morbidity in SAC [20]. The programme has been utilizing trained primary school teachers to conduct MDA. The Ministry of Health (MoH), Kenya has adopted a community health strategy following the WHO recommendation for enhanced prevention and control of NTDs towards elimination [21,22]. In this strategy, MDAs are conducted at the community level by community health volunteers (CHVs) (lay health workers) at household level also known as door-to-door distribution [22]. These individuals are ‘volunteers’ for various programmes and do not receive remuneration for their annual work commitment except incentives for individual projects during the interventions. This notwithstanding, parents/guardians with children below 5 years of age have free access to post-natal care in public health facilities called *Malezi Bora* meaning good nutrition [23]. The *Malezi Bora* events are organized bi-annually to increase utilization of routine health and nutrition services [23]. Given the available options, we conducted this study to identify the preferred drug delivery approaches for pilot roll-out of the new paediatric formulation by the community members and other stakeholders to inform the programme.

## Methods

### Study site

This mixed methods study was conducted in May 2022 in two schistosomiasis-endemic counties of Kenya located approximately 500 miles apart (Homa Bay and Kwale). Kwale County is located 30 kilometres (kms) southwest of Mombasa in coastal Kenya, with an area of 8360 km^2^ and an estimated population of 866,829 persons [24]. Homa Bay County is situated in western Kenya, bordering Lake Victoria basin which is endemic for *S*. *mansoni*.

### Study design and setting

Using quantitative and qualitative methods, we conducted a cross-sectional study in two purposively selected counties, owing to previous studies showing high prevalence of schistosomiasis [25,26]. The lower administrative sub-region called sub-county was also purposively sampled, and four villages were randomly selected in each county for sampling of both quantitative and qualitative participants. In each village, households with PSAC were mapped by both the research team and CHVs. Systematic random sampling method selected the household to be included in the quantitative survey, and the respondents were parents/guardians of PSAC. The first household was randomly selected and the next 4^th^ household with PSAC followed systematically to the right until the required sample size was reached. In the event the sample size was not reached, the neighbouring village was included. Each study participant was interviewed using a pre-tested questionnaire to capture information on socio-demographics, preferred drug delivery approaches/strategies and suggestions on the planned MDA roll-out.

In the qualitative arm of the study, participants were recruited also from the four villages after questionnaire survey was conducted. However, only parents/guardians of PSAC whose households were not enrolled for the quantitative survey were recruited for focus group discussions (FGDs). In addition, FGDs were also conducted with CHVs recruited from the same villages. Also, key informant interviews (KIIs) were conducted with opinion leaders and health care workers recruited from the same area. All participants were purposively selected based on their availability and suitability to enhance understanding of the study. Interviews were conducted using interview guides to capture perceptions, opinions and suggestions on drug delivery approaches for the planned roll-out of the new drug. All interviews were conducted in local language (Dhuluo and Dingo), Swahili or English depending with understanding and preference of the study participants. Before data collection, community meetings were held with village and ward administrators and area chiefs to explain the study’s purpose, procedures, potential risks, and benefits. Before the interviews, the study was explained to each participant, and written informed consent forms were signed by all study participants.

### Study population and sample size

The focal population was parents/guardians of PSAC drawn from four randomly selected villages in each county for the quantitative survey. A structured questionnaire was created and administered, including data on socio-demographics, preferred methods on drug delivery and suggestions on how the MDA can be implemented. For the quantitative survey, the sample size was calculated using the Cochran formula [27]; n  =  Z^2^ pq/e^2^; where Z is the score for a 5% type 1 error for a normal distribution (Z  =  1.96), p is the assumed prevalence of schistosomiasis in PSAC taken as 50%. This assumed prevalence (50%) was used since no similar study had been conducted in the area previously. Using a margin of error (e) of 4.5, the final estimated sample size was 384 households with PSAC. However, the study adjusted the figures with the design effect (DE) of 1.2 for Kwale and 0.75 for Homabay to increase the sample size to 748 which was distributed into the two sub counties based on the formula below:

$$n=\frac{z^{2}p(1-P)}{d^{2}}*DE$$

In the qualitative data collection, CHVs familiar with the selected villages helped to mobilise adult participants (18 years old and above) who were purposefully selected. Further screening was done to ensure that all the participants met the inclusion and exclusion criteria, ensuring they were a true representation of their villages before seeking their informed consent [28]. Twelve FGDs were conducted with parents/guardians of PSAC in both counties, segregated by age and gender. In addition, seven FGDs were conducted with purposively selected CHVs drawn from the selected village. More so, purposively selected KIIs were conducted with 28 healthcare workers (clinical officers, nurses, public health officers, community health extension workers and sub-county NTD program personnel) and 17 opinion leaders (community leaders) in both counties. The saturation model was used to determine the number of FGDs and KIIs to be conducted in each study area [29].

### Community mobilization and household survey

Three days before commencing the study, the study team held meetings with the county, sub-county leaders and village elders at the community level to sensitize them about the study. During the sensitization period, study participants were informed to expect research assistants accompanied by CHVs at their homesteads for data collection. Data collection was done by trained research assistants using android-based mobile phones in which a pre-tested questionnaire which had been programmed into Open Data Kit (ODK) software [30]. Socio-demographic data, information on the preferred method of drug distribution, preferred timings of the distribution and suggestions on the planned roll-out of the new drug were obtained during the interviews.

### Focus group discussions and key informant interviews

We collected data using interview guides for each category by trained moderators and note takers using digital voice recorders. The purpose was to gather information on the respondent’s preferred method of drug delivery, previous experiences with similar programmes and suggestions for the planned MDA. Data was audio-recorded, transcribed and simultaneously translated into English during transcription then coded according to pre-determined themes of the study. The notes of all the transcripts were typed and saved in MS Word.

### Statistical quantitative data analysis

Survey data was captured electronically using the Open Data Kit (ODK) system and imported into STATA version 15.1 (STATA Corporation, College Station, TX, USA) for cleaning, processing and analysis. Descriptive statistics were calculated for the independent variables which included socio-demographic and socio-economic characteristics and parent/guardians’ preferred methods of drug delivery. Socio-demographic and socio-economic factors included age, gender, number of children, educational level, marital status, religion, occupation and family member who decides on health matters. These variables were assessed using frequencies and proportions for the categorical variables and means and standard deviations for the quantitative variables. The preferred methods of drug delivery approaches were analyzed using frequencies, proportions and percentages.

### Qualitative data analysis

All notes were imported to Nvivo version 12 software for further processing and data analysis. The software facilitated distinguishing themes and sub-themes by running queries, identify patterns within the coded data. The coding process involved a critical review of the transcripts and notes to identify emerging and sub themes. For specific analysis, advanced and straightforward coding queries were used to search for words and phrases in the notes. A framework approach was applied to identify emerging themes from the interviews iteratively up to the point of saturation and aligned with pre-determined themes [29]. Triangulation for the two data sets was used to complement each other and further explore between each of the data set. A code book is listed below to guide on the coding of KIIs and FGDs.

### KII code book

**H-KII-OL**: Homa Bay KII opinion leader; **K-KII-OL**: Kwale KII opinion leader; **H-KII-HC**: Homa Bay KII Health care workers; **K-KII -HC: Kwale KII** Health care workers.

### FGDs code book

**H-FGD-M:** Homa Bay FGD male; **H-FGD-F**: Homa Bay FGD female; **K-FGD-M**: Kwale FGD male; **K-FGD-F**: Kwale FGD female; **H-FGD-CHV**: Homa Bay FGD community health volunteers; **K-FGD-CHVs**: Kwale FGD community health volunteers.

### Ethical clearance

Ethical clearance was received from the Kenya Medical Research Institute (KEMRI), Scientific and Ethics Review Unit (SERU No. 4992) and ethical committee at the Technical University of Munich (TUM) (731/21 S). Written informed consent was sought from all study participants. In addition, written informed consent was sought from study participants of the qualitative arm of the study with specific mention of the audio recording process for the FGDs and KIIs. Participant names were replaced with alphanumeric unique identifiers to ensure pseudonymity and confidentiality.

## Results

### Socio-demographic and socio-economic characteristics of the participants

In the quantitative survey, 690 parents/guardians of PSAC participated in this study. Majority of the participants were women, n = 594 (86.1%), with a mean age of 34.1 (SD = 11.3, min-max = 18–86). Most of the participants were married or in a relationship n = 599 (86.8%), with no education n = 297 (43.0%) and were housewives, n = 202 (29.3%). In most of the households, women (wives or mothers) n = 423 (61.3%) were the main decision makers in matters regarding health in the family (Table 1). All the details of county stratifications are in shown in Table 1.

**Table 1:** Socio-demographic and socio-economic characteristics of participants.

Factor	Overall; n (%)	Homabay; n (%)	Kwale; n (%)
**Age**			
Mean (Min-Max)	34.1 (18–86)	33.3 (18–86)	34.6 (18–74)
**Gender**			
Female	594 (86.1%)	230 (84.6%)	364 (87.1%)
Male	96 (13.9%)	42 (15.4%)	54 (12.9%)
**Number of children**			
Mean (Min-Max)	2 (1–7)	2 (1–5)	2 (1–7)
**Marital status**			
Single	20 (2.9%)	9 (3.3%)	11 (2.6%)
Married/In a relationship	599 (86.8%)	232 (85.3%)	367 (87.8%)
Widowed/Divorced	61 (8.8%)	31 (11.4%)	30 (7.2%)
Separated	10 (1.5%)	0 (0%)	10 (2.4%)
**Educational level**			
No education	297 (43.0%)	75 (27.6%)	205 (49.0%)
Primary	281 (40.7%)	106 (39%)	175 (41.9%)
Secondary	75 (10.9%)	49 (18.0%)	26 (6.2%)
Post-Secondary	37 (5.4%)	25 (9.2%)	12 (2.9%)
**Religion**			
Christian	331 (48%)	268 (99%)	63 (15.1%)
Muslim	354 (51.3%)	1 (0.4%)	353 (84.5%)
Others	5 (0.7%)	3 (1.1%)	2 (0.5%)
**Occupation**			
No work	63 (9.1%)	22 (8.1%)	41 (9.8%)
Farming/Fishing	181 (26.2%)	68 (25.0%)	113 (27.0%)
Civil servant	18 (2.6%)	9 (3.3%)	9 (2.2%)
Casual worker	14 (2.0%)	10 (3.7%)	4 (1.0%)
Housewife	202 (29.3%)	53 (19.5%)	149 (35.7%)
Private employment	13 (1.9%)	8 (2.9%)	5 (1.2%)
Small scale enterprise	172 (24.9%)	88 (32.4%)	84 (20.1%)
Student	8 (1.2%)	6 (2.2%)	2 (0.5%)
Others	19 (2.8%)	8 (2.9%)	11 (2.6%)
**Family member making decision regarding health matters**			
Men (husbands, fathers)	205 (29.7%)	62 (22.8%)	143 (34.2%)
Women (wives, mothers)	423 (61.3%)	185 (68.0%)	238 (56.9%)
Children	1 (0.1%)	1 (0.4%)	0 (0%)
Self (the sick person)	1 (0.1%)	0 (0%)	1 (0.2%)
Grandparents/aunties/uncles	46 (6.7%)	16 (5.9%)	30 (7.2%)
Religious leaders	1 (0.1%)	0 (0%)	1 (0.2%)
Others	13 (1.9%)	8 (2.9%)	5 (1.2%)

### Preferred methods of drug delivery

Overall, the survey results indicated that the preferred method of drug delivery among the participants was community-based MDA (cMDA) 598 (86.7%), followed by health facility/fixed points (HF/FP) 398 (57.7%), Malezi Bora/Child Health Day (CHD) at 85 (12.3%) and the least was other methods at 45 (6.5%; multiple answers possible) (Table 2). We noted that after stratification by age groups, gender, marital status, occupation, education level and religion, community MDA was still the most preferred method of drug delivery in all profile categories (Table 2).

**Table 2:** Reported preferred methods of drug delivery by socio-demographic characteristics.

Factor / Delivery method	CHD	HF/FPD	sMDA	cMDA	All methods
**Overall**	85 (12.3%)	398 (57.7%)	142 (20.6%)	598 (86.7%)	45 (6.5%)
**Age group**					
≤20	4 (0.6%)	16 (2.3%)	3(0.4%)	25 (3.6%)	1(0.1%)
21–30	34(4.9%)	182(26.4%)	62(9.0%)	241(34.9%)	20(2.9%)
31–40	32(4.6%)	124(18%)	39(5.7%)	202(29.3%)	16(2.3%)
41–50	9(0.6%)	47(6.8%)	21(3.0%)	79(11.5%)	5(0.7%)
>50	6(0.9%)	29(4.2%)	17(2.5%)	51(7.4%)	3(0.4%)
**Gender**					
Male	13(1.9%)	46(6.7%)	15(2.2%)	82(11.9%)	3(0.4%)
Female	72(10.4%)	352(51.0%)	127(18.4%)	516(74.8%)	42(6.1%)
**Marital status**					
Single	2(0.3%)	12(1.7%)	5(0.7%)	16(2.3%)	1(0.1%)
Married	71(10.3%)	340(49.3%)	118(17.1%)	523(75.8%)	39(5.7%)
Widowed/Divorced	9(1.3%)	39(5.7%)	15(2.2%)	51(7.4%)	3(0.4%)
Separated	3(0.4%)	7(1.0%)	4(0.6%)	8(1.2%)	2(0.3%)
**Occupation**					
No work	13(1.9%)	45(6.5%)	22(3.2%)	54(7.83%)	7(1.0%)
Farming/Fishing	16(2.3%)	88(12.8%)	27(3.9%)	151(21.9%)	7(1.0%)
Civil servant	3(0.4%)	11(1.6%)	7(1.0%)	17(2.5%)	2(0.3%)
Casual worker	0(0.0%)	5(0.7%)	1(0.1%)	13(1.9%)	0(0.0%)
Housewife	23(3.3%)	118(17.1%)	37(5.4%)	179(25.9%)	16(2.3%)
Private employment	3(0.4%)	9(1.3%)	4(0.6%)	11(1.6%)	1(0.1%)
Small scale enterprise	26(3.8%)	111(16.1%)	39(5.7%)	148(21.5%)	12(1.7%)
Student	0(0.0%)	3(0.4%)	0(0.0%)	7(1.0%)	0(0.0%)
Others	1(0.1%)	8(1.1%)	5(0.7%)	54(7.8%)	0(0.0%)
**Education**					
No education	15(2.2%)	154(22.3%)	29(4.2%)	258(37.4%)	5(0.8%)
Primary	54(7.8%)	174(25.2%)	76(11.0%)	242(35.1%)	33(4.8%)
Secondary	13(1.9%)	47(6.8%)	17(2.5%)	64(9.3%)	4(0.6%)
Post-Secondary	3(0.4%)	23(3.3%)	13(1.9%)	34(4.9%)	3(0.4%)
**Religion**					
Christian	31(4.5%)	215(31.2%)	80(11.6%)	289(41.9%)	12(1.7%)
Muslim	53(7.7%)	179(25.9%)	61(8.8%)	307(44.5%)	32(4.6%)
Non-practicing	1(0.1%)	4(0.6%)	1(0.1%)	2(0.3%)	1(0.1%)

### Platforms/Methods of drug distribution

In the qualitative analysis, the majority of the study participants felt that the drug should be distributed through cMDA, also referred to as the door-to-door method by the CHVs, since they are familiar with the villages and households with PSAC. CHVs were considered to be able to explain and communicate the effects of the new drug. Other reasons were that given their young age, some children targeted in the new drug campaign are not yet enrolled in school, and the CHVs know the households with children below five years of age and community members trust them.

“*Some children are not found in school like those aged 1–2 years*. *Therefore*, *there is need for the CHVs to walk in the community*. *Again*, *depending on the mechanisms used by the ministry*, *other community members may be involved for door-to-door distribution”* (FGD Male parent/guardian, Homa Bay).

Nevertheless, some people in both interview categories (KIIs and FGDs) in the two counties felt that given CHVs have no medical training, they may not be suitable for distributing the drug. These participants suggested that healthcare workers would be more appropriate since they are trained, have more information about the new drug and can attend to any adverse drug reactions. Below is one of the recommendation from participants:

“*The drug should be taken to the health centre*. *I do not trust CHVs*. *I want my child to be attended by a professional and not CHVs*. *Health days in hospitals are okay but not CHVs*.*”* (KII opinion leader, Homa Bay).

Some of the study participants, mainly in the health care category, agreed and suggested that healthcare workers were more appropriate for paediatric drug distribution given their medical skills. These individuals felt that the drug should be available at health facilities for PSAC parents/guardians to access during clinic days. Other reasons for using health facilities were that they tended to have adequate storage for the drugs, that many people visit health facilities seeking treatment, and that trained healthcare workers can keep proper records of the drugs dispensed. That notwithstanding, study participants felt that using health facilities will have challenges, especially workflow and overcrowding. Below is a selected extract from the interviews:

“*In hospitals*, *you need a person who will do the dispensing of drugs as well as recording of the treatment information*. *The only problem at the health facility will be workflow because there will be many people in and out of the hospital*.*”* (KII HCW, Kwale).

Participants from the health workers category felt that using schools would also be another way to increase the coverage since most schools are accessible and some of the targeted children are already enrolled in school. Other reasons given for using schools were that there would be close supervision, the teachers are authority figures and are trusted by the community members. In addition, there is existing NSBDP framework in which teachers, who distribute drugs to pupils, are also involved in increasing awareness of schistosomiasis amongst children and by extension, to the parents/guardians of PSAC. Moreover, others argued that children are at risk of getting other infections if taken to health facilities.

“*For increased coverage*, *I would suggest you use schools because most of them are accessible and some of the target age group is also in school as opposed to them coming to a facility because most of the time in hospitals*, *children are at risk of getting other infections*.*”* (KII HCW, Kwale).

A few healthcare workers category in both counties, felt that integration of the schools Early Childhood Development (ECDs), maternal child health (MCH), and outreaches normally done in social gatherings by CHVs during *Malezi Bora weeks* could work for the targeted age group. The reason given was that parents/guardians stop attending post-natal care once a child gets the measles vaccine which is routinely administered at nine months, unless the child is sick.

“*I tend to feel*, *because you are targeting the under-five*, *we can take advantage of the MCHs/the ECDs and have outreaches to get those people because I know most women*, *when they finish measles vaccines*, *we rarely see them again*. *They only get back to the facility when their child is sick*. *So*, *with that group we must go back to the schools or outreaches*, *so those three methods might yield*.*”* (KII HCW, Kwale).

### Timing and frequency of drug distribution

Study participants in both counties felt that careful consideration of distribution timings would be essential as community members go to the field to cultivate crops during the rainy seasons. While during the hot/dry season, community members go to fetch water for domestic use considering most houses lack piped water. The preferred times for distribution were the morning hours or at the end of the day when parents and guardians of PSAC can be found in the homesteads. In addition, participants recommended that drug distributors should be given enough time to reach out to every target recipient considering that not all will be available on the first distribution day. Also, there were suggestions that the drugs should be given in three-month intervals, availing enough supplies before the actual day of distribution for proper coordination and timely distribution.

“*The treatment has to be done frequently because you are going to visit today*, *it is not guaranteed that you will find everyone at home*, *so if you miss them today*, *maybe the other round you will get them”* (KII HCW, Homa Bay).

### Measures for effective drug distribution

One of the suggestions given by study participants, especially in the KIIs and FGDs in the health care category, was that the programme should consider providing CHVs with labelled T-shirts, which could be a way of continued sensitization during the drug distribution period. CHVs participants felt that if they were involved in drug distribution, it would be necessary to give them monetary compensation to motivate them to work more efficiently and effectively. The involvement of community leaders was also suggested as a method that can make the programme effective by building confidence in the community.

“*It would be good for the project to involve the local structures… let the chiefs be supported with lunch and airtime because it involves a lot and once you have involved the chief*, *they do a fantastic job*. *If you give them lunch*, *transport and airtime that’s enough for them*. *The other person I would prefer to be included is the village administrator*, *those are very key people who can mobilize a whole unit*. *You know village units are almost like a location–they are very important people who can do a fantastic job together with CHVs”* (KII HCW, Kwale).

Study participants mostly drawn from the parents/guardians FGD category and opinion leaders in Homa Bay County, felt that children should be given food before they take the drug since from their experience, children are provided with some snacks before swallowing PZQ. In addition, they felt that the programme could provide food to the children before swallowing the drug.

“*From the previous campaign*, *it was known that you were supposed to eat before taking the drug*. *We would wish that the ministry to provide at least some food for the children before taking the drug”* (FGD Male, Homa Bay).

## Discussion

Our findings show that most of the study participants preferred the community-wide MDA, also called the door-to-door method of drug distribution. Survey results indicated that 598 (86.7%) respondents preferred the distribution of the medication through community MDA. In this scenario, CHVs are responsible for the safe distribution of the drugs, motivating communities to receive treatment, creating and updating treatment registers, monitoring side-effects and compiling treatment coverage reports [31]. Parents/guardians in both counties may prefer this drug delivery method due to the convenience of their children receiving the treatment at the household level without having to make efforts to travel for sometimes long distances and possibly incur transport costs to health facilities. A study in Ghana on determinants of health-seeking behaviour for schistosomiasis found that only 20% of schistosomiasis-related signs/symptoms were reported to health facilities [32] perhaps meaning that for various reasons, people failed to visit health facilities and would go only when symptoms turned severe. Other reasons could be that people are reluctant to visit health facilities when they are clinically well, and they might be too busy to take their children to receive the treatment considering that they are occupied by their daily household chores or income-generating activities.

A previous study has shown that using CHVs may be a viable distribution strategy to consider in Kenya, alongside the ongoing school-based treatment strategy, as the national control programme intensifies activities toward the control and elimination of schistosomiasis [33]. At the programme level, it is worth noting that this method of drug distribution presents many advantages like increased coverage, traceability of children, facilitation of receiving consent, and that CHVs are well known and trusted by the communities [31]. However, the new drug dosage might be calculated using weight instead of height, which uses a dose pole. If the programme adopts the community MDA strategy, CHVs will have to walk with weighing scales from house to house which could bring logistical challenges of calibration and accuracy on the dosage due to the frequent movement of the weighing scales.

Other possible reasons for participants preferring community MDA could be that parents/guardians are hesitant to visit health facilities, especially if the child is not sick, for fear of their children being exposed to infections. In addition, schistosomiasis is not a major life-threatening disease with mild symptoms at early stages which may cause people to delay seeking healthcare. A study done in West Africa by Danso-Appiah *et al*., (2010) on schistosomiasis showed that healthcare seeking was relatively lower for patients with chronic symptoms, with perceived severity as the main predictor for visiting a health facility [34].

Just over half of study participants (n = 398 (57.7%) indicated that they would prefer the new drug to be dispensed at the health facility or at fixed points. The main reason cited was CHVs’ lack of clinical training and capacity to attend to possible drug reactions like side effects or severe adverse reactions. A study by Parker and Allen in 2011 on MDA compliance in Uganda found that the success or failure of MDA depended on many reasons like relations between drug distributors and targeted groups, rumours and conspiracy theories about the ‘real’ purpose of treatment and subjective experiences of side effects from treatment [35]. These factors may hinder drug distribution and foster mistrust in the community towards CHVs and the programme [36]. Senyonjo *et al*. (2016) found that the lack of support from health staff for handling potential adverse events was a de-motivating factor for NTDs volunteers [37].

Studies on the importance of training CHVs have shown that without proper training, NTDs volunteers could not perform their duties well in terms of educating participants or dealing with questions that arose during drug distribution [38,39]. This indicates the need for careful training of the CHVs who might be involved in drug distribution in future interventions. Dabo *et al*., (2013) found that higher treatment coverage rates were achieved when formal health workers were present at the time of distribution [40].

In the current programme, health facilities would present many advantages in the event weight will be used for dosing. This is because some health facilities have the weighing scales in place which are used routinely and also their movement will be reduced for more accurate dosage calculations. That notwithstanding, there might be concerns of overcrowding and understaffing in the health facilities considering that routine health activities will continue throughout paediatric schistosomiasis treatment campaigns. This may cause strain on healthcare workers who frequently face understaffing [32]. This will require the programme to engage additional staff at the times of drug distribution who might need to be trained and remunerated.

Other reasons given for preferring distribution through the health facility were good supply chain management and the linkage of storage and community implementation units to health facilities. CHVs would still need to be involved in mobilizing community members to take their children to health facilities for treatment. Meaning that most community health programmes/interventions in the country might be dependent on CHVs for better outcomes. Drug delivery approaches using CHD were rated low by parents and guardians with only 12.3% indicating preference for this platform. This could be because community members know about *Malezi Bora* and the term CHD is commonly used by the healthcare workers. Also, the research team equated *Malezi Bora* as facility based. Hence use of this platform would call for continued social mobilization and awareness building around CHDs by the concerned health departments.

Our results show that only a few healthcare workers opted for distribution in ECDs, citing that some of the targets recipients of the drug are not yet enrolled in ECDs. Similar studies have shown that the area/region of MDA recipients is important for increased compliance [35,41]. Nevertheless, participants suggested that the schools and SAC can be used to communicate with the parents and guardians and their younger siblings about the programme. It is worth noting that the NSBDP in Kenya has successfully trained primary school teachers to conduct school-based MDA [42]. This might present a good leverage for the programme to mop up or act as a fixed point in the event CHVs distribute the drugs.

The timing of the MDA may influence the programme’s success through increased coverage. In the current study, parents and guardians particularly in Kwale County preferred drug distribution to occur during the morning hours in the dry season. A study by Omedo *et al*., (2012) found that in Western Kenya, drug distribution for schistosomiasis control programmes was dictated by the harvest season, during which people had access to food with which to take the drugs to reduce side effects [33]. A separate study on MDA for NTDs in Ghana showed that drug distribution occurred at the same time as the cocoa harvesting season, hindering the program and prolonging the period of the deworming activity, which requires additional resources [34]. This calls for the programme’s deliberate coordination and planning to approach drug distribution in tune with the ongoing community activities. It is worth noting that there were minimal contextual differences amongst respondents in the two counties. As things currently stand, there is need to consider whether the programme adopts directly observed treatment (DOTs), and whether children will receive food/snacks before taking the drug.

### Study limitations

A key limitation of the study was including predominantly mothers in the sampling when it is known that fathers are also key-decision makers regarding child health. Hence, further exploration should be undertaken with the fathers to know their views.

## Conclusion

The findings from this study show that the preferred distribution strategy for the new drug was cMDA due to its convenience for most participants. The health facility or fixed points were the second most preferred strategy by the study participants. Points in favour of facilities were that dispensing of the new drug would be done by trained health care workers who can attend to the children in the event of any adverse drug reaction and side effects. However, a combination of delivery approaches could be considered depending on the local context, special circumstances and during mop-ups to achieve the desired outcome. This study also underlines the importance of adequate planning, consultation, coordination, proper timings and community involvement prior to delivery of the new drug for better outcomes independent of the delivery approach used.
