# Correction: Community preferred drug delivery approaches for pilot roll-out of a potential novel paediatric schistosomiasis treatment option in two endemic counties of Kenya: A mixed methods study

**DOI:** 10.1371/journal.pgph.0005653

**Published:** 2025-12-15

**Authors:** Janet Masaku, John M. Gachohi, Alice Sinkeet, Mary Maghanga, Florence Wakesho, Wyckliff Omondi, Nora Monnier, Peter Steinmann, Lisa Sophie Reigl, Isabelle L. Lange, Andrea S. Winkler, Sammy M. Njenga, Mary Amuyunzu-Nyamongo

There are errors in the Funding statement. The correct Funding statement is as follows: The study received funding from Merck KGaA, Darmstadt, Germany, and is part of the EDCTP2 programme supported by the European Union (grant number RIA2019-2895-ADOPT). The funders had no role in the study design, data collection and analysis, preparation of the manuscript or the decision to publish.
